# Optimized animal model to mimic the reality of stress-induced depression in the clinic

**DOI:** 10.1186/s12888-017-1335-x

**Published:** 2017-05-06

**Authors:** Yi Zhang, Yuting Wang, Hui Lei, Lei Wang, Liang Xue, Xin Wang, Xiongzhao Zhu

**Affiliations:** 1 Medical Psychological Center, The Second Xiangya Hospital, Central South University, 139 Renmin Road, Changsha, Hunan 410011 People’s Republic of China; 2 Department of Clinical Psychology, Maternal and Child health Hospital of Hunan Province, Changsha, Hunan 410008 China; 3 College of Education, Hunan Agriculture University, Changsha, 410128 People’s Republic of China

**Keywords:** Depression, Animal model, Latent profile analysis, Percentile method, Cutoff

## Abstract

**Background:**

Animal models are useful tools for verifying the relationship between stress and depression; however, an operational criterion for excluding the resilient animals from the analysis has not been established yet, which hinders the model’s ability to more accurately mimic the scenario in humans.

**Methods:**

To induce depression-like symptoms, rats received maternal deprivation (MD) during PND1–14, and/or chronic unpredictable stress (CUS) exposure. The latent profile analysis (LPA) was used to determine latent subgroups in treatment naive adult rats. The percentile method was used to distinguish sensitive and non-sensitive behaviors in rats.

**Results:**

The sucrose preference rate of treatment naive adult rats was fit using a Beta distribution, while immobility time was fit using a Gamma distribution. Indexes of behavioral tests revealed the 4-class model as the best fit for treatment naive adult rats. The incidence of stress-resilience in MD rats was significantly higher than that in CUS rats and MD + CUS rats. There was a significantly higher incidence of stress-resilience in CUS rats compared with MD + CUS rats. Recovery rate of anhedonia-like and sub anhedonia-like behaviors in CUS rats was significantly higher than that in MD and MD + CUS rats. There was a significantly higher recovery rate of anhedonia-like behaviors in MD rats compared to MD + CUS rats.

**Conclusions:**

The percentile method is suitable for setting up an operational cutoff to classify depression-like, sub depression-like, and resilient behaviors in rats exposed to MD and CUS.

## Background

Depression is a complex, heterogeneous, and often chronic psychiatric disorder [1, 2]. In the clinic, depression exhibits various clinical phenotypes and inconsistent responses to current antidepressant therapy [3]. Currently, depression is viewed as a multifactorial disease that encompasses environmental, social, psychological, and biological factors. Among these etiologies, psychological stress is one of the most relevant risk factors for psychopathologies, and the exposure to stressful events powerfully triggers depressive episodes [4]. However, ethical limitations prevent the validation of the stress hypothesis and the causality between stress and depression in humans. Animal models are therefore important for investigating the molecular and cellular mechanisms as well as treatment response to antidepressant agents in different subtypes of depression.

It is normally acknowledged that the same stress only causes psychopathology in a small portion of the population, and only the patients with obvious symptoms receive treatments or intervention. Our previous study observed that some animals are resilient and can cope with the applied stressors, while some are much more vulnerable or susceptible to stressors and drop into an anhedonia and/or despair-like statue [5]. However, the animals that are not sensitive to stress are also included in the analysis, which is not the scenario in the clinic. To mimic the scenario of depression development in humans, animal models should also exclude animals not sensitive to stresses. An operational criterion needs to be established for depressive behaviors to distinguish between animals that are sensitive to stress from those that are not sensitive to stress.

The involvement of stress in the development of depression has been widely investigated in animals. For example, animal models established with maternal deprivation and chronic unpredictable stress-exposure are widely used models that mimic stress experienced in early life and during adulthood of humans, respectively [6, 7] and the validity of these depression models have been widely verified [8, 9]. Due to patients with depression who have experienced stresses in both their early life and adulthood, the combination of these two stress models may better reflect the multiple stresses-induced depression in human. However, how an animal model of depression mimics the responses exhibited by humans where only stressor-sensitive individuals can develop depression has not been well addressed although some studies may actually exclude animals not sensitive to stresses [10, 11]. Moreover, different animals, different stresses, and different behavioral test methods may yield different readouts in behavioral tests. A universal approach to establish an operational criterion for depressive behaviors to exclude behaviorally abnormal animals and animals not sensitive to stresses may improve the current animal models of depression.

The percentile method is used in medicine to determine reference limits [12–14]. The primary advantage of this method is that the reliability of sample percentiles can be determined without making any assumptions about the distribution shape (Gaussian or Skewed distribution) of the population. Determining the percentiles of a sample is required for the exclusion of specified population percentages (generally, more than 5%, less than 15%) [12, 14]. However, whether the percentile method could be used to determine the cutoff value of depressive behaviors in animals has not been validated. In this study, we first performed a latent profile analysis (LPA) to see whether latent subgroups can be identified in a large sample of untreated adult rats using the sucrose preference rate and immobility time. If it is successful, we then determined a cutoff value of anhedonia-like and despair-like behaviors using a percentile method depending on readouts of behavioral tests, followed by analysis of the incidence of different behavioral intensity and the effect of escitalopram treatment.

## Methods

### Animals

**Fig. 1:**
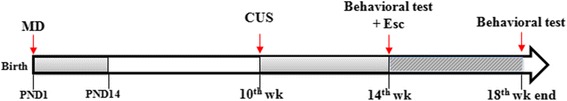
Scheme of experimental design. During the early post-natal phase (PNDs 1–14), pups in MD and MD + CUS group received maternal deprivation (MD). At *10th weeks*, chronic unpredictable stress (CUS) procedure began. At the end of *14th weeks*, depression-like and sub depression-like rats, defined by the operational cutoff of behavioral test readouts, were randomly grouped into escitalopram treatment and saline. The sucrose preference test was carried out every week from *9th* to *18th weeks*. Forced swimming test was performed before 1 week and at the end of the CUS procedure as well as at the end of antidepressant treatment. CUS was still exposed during antidepressant treatment in order to maintain the depressive-like behavior

Pregnant adult Sprague–Dawley rats (SLAC Laboratory Animal, Shanghai, China) were housed individually in plexiglass cages and checked daily until delivery. Animals were kept on a 12-h light/dark cycle (lights were switched on at 08:00) with food and water ad libitum, and constant temperature (22 ± 2 °C) and humidity (50–55%). The day of delivery was designated postnatal day 0 (PND 0). Newborn male pups were randomly assigned to one of the following 4 manipulations: Pups that received only maternal deprivation from PND1 to PND14 (MD, *N* = 111), only chronic unpredictable stress from 10th week (CUS, *N* = 122), and both maternal deprivation from PND1 to PND14 and chronic unpredictable stress from 10th to 14th week (MD + CUS, *N* = 99) in addition to a control with no experimental stress (NOR, *N* = 120). All rats were weaned at PND 21 and housed in groups of two to three. In addition, 309 treatment native adult Sprague–Dawley rats (10 weeks) were purchased from SLAC Laboratory Animal. The study design is shown in Fig. 1.

### Maternal deprivation (MD)

The MD paradigm was designed as previously described [5]. Briefly, pups were separated from their mothers for 6 h daily from PND 1 to PND 14 (the separations occurred at 9:00–15:00). To block communication between littermates, each offspring was placed in a single cell (a cage with a size of 32 cm × 32 cm × 14 cm was divided into four cells of the same size) covered with dry sawdust. At the end of the separation period, pups were returned to their maternal cages. Temperature inside the cell was maintained at 32 °C for the first five deprivations and 30 °C for the other deprivations.

### Chronic unpredictable stress (CUS)

The CUS paradigm was modified from a previously established protocol [5]. Briefly, rats at 10 weeks old were exposed to a variety of sequential stressors for 4 weeks: electric foot shock for 20s (0.6 mA, 1-s duration, average 1 shock/10s), an elevated open platform (10 cm × 10 cm, 160 cm in height) for 2 h, crowding for 10 h (5–6 rats cage), wet bedding for 15 h, water deprivation for 24 h, food deprivation for 24 h, and restraint stress for 2 h. In order to establish unpredictability, stressors were distributed randomly every day at different times.

### Antidepressant treatment

In this study, the widely used antidepressant escitalopram (Esc) (H. Lundeck A/S Copenhagen, Denmark) was applied, and the protocol was conducted as previously described [6]. Escitalopram was dissolved in 0.9% physiological saline (1 mg/ml) immediately before use; then, it was administered intraperitoneally and daily (5 mg/kg) in the morning over a 4-week period of time for depressive rats (*n* = 20 each group). Naïve rats (*n* = 20) received saline as a vehicle injection. In order to maintain the depressive-like behavior, CUS was still exposed during the antidepressant treatment.

### Sucrose preference test (SPT)

Sucrose preference test was performed as described previously [5] to assess the hedonia level in rats. This test was carried out before CUS and at the end of each week. Rats were kept individually in separate cages, and were allowed to adapt to two bottles of solution (filled with 1.5% sucrose solution) for 24 h. For the next 24 h, one bottle of sucrose solution was replaced with water. Then, the rats were subjected to 18 h of food and water deprivation, followed by exposure to two pre-weighed bottles of solution (1.5% sucrose solution and plain water, respectively) for 1 h. The position of the bottles was switched. After the test, the weight of sucrose solution and water consumed was recorded. Sucrose preference was calculated as a ratio of the weight of sucrose solution consumption to the weight of total fluid intake.

### Forced swimming test (FST)

FST was carried out to measure behavioral despair following a previously established protocol [5]. The test was performed at the beginning and at the end of the CUS procedure, as well as at the end of the antidepressant treatment. Two swimming sessions were conducted: a 15-min pretest on day 1 followed by a 5-min test the next day. Rats were forced to swim in a glass cylinder (21 cm diameter × 46 cm high) containing 30 cm depth of water (25 °C). Behavior was video-recorded using SMART video-tracking system (Panlab, Spain). The immobility time (time a rat spent in floating with no active activity other than those necessary to keep head above the water) was analyzed. Water was changed after each test.

### Statistical analysis

Data analysis, excluding latent profile analysis (LPA), was performed using the statistical software SPSS 17.0. LPA was performed using Mplus version 7.0. First, the distribution of indexes in behavioral tests was analyzed. Then, exploratory LPA was performed to identify latent subgroups based on the behavioral indexes of treatment in native adult rats. The model selection and comparison were conducted by evaluating the fit of a 1-class model; the number of latent classes was then increased until the addition of latent classes was not justified. Five analyses were used to determine the optimal number of class: 1) the Akaike Information Criterion (AIC), Bayesian Information Criterion (BIC), and Adjusted BIC (aBIC) values were calculated with lower values indicating a better fit; 2) The *P* value of Lo-Mendell-Rubin-adjusted likelihood ratio test (A LMR) was used to determine whether more classes (*P* < 0.05) or fewer classes (*P* > 0.05) fit better; and 3) A standardized measure of entropy was used to assess classification uncertainty based on model-based posterior probabilities with higher value indicating better classification of individuals. After determining the best class solution, indexes of behavioral tests were compared across the latent classes using the one-way analysis of variance (ANOVA) [15]. The percentile method was used to determine the operational cutoffs for identifying depressive and sub-depressive behaviors. Chi-square test was used to compare the incidence of depressive behaviors. A *P* < 0.05 was considered statistically significant.

## Results

### The distribution of sucrose preference rate and immobility time in treatment naive adult rats

**Fig. 2:**
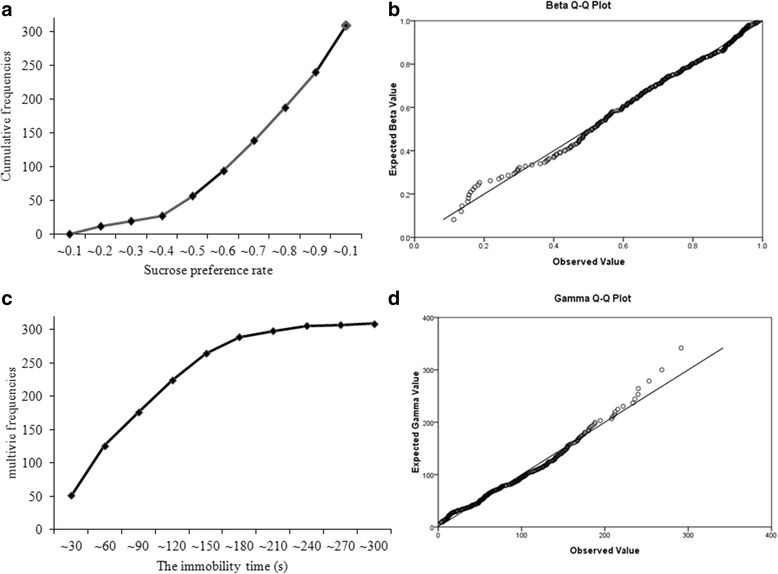
Cumulative distribution and Q-Q plots for the sucrose preference test (SPT) and forced swimming test (FST) in treatment native adult rats. Cumulative distribution **a** and Beta Q-Q plot **b** for sucrose preference rate from SPT in treatment native adult rats; Cumulative distribution **c** and Beta Q-Q plot **d** for immobility time from FST in treatment native adult rats. *N* = 309

Behaviors of treatment naive adult SD rats (*N* = 309) were measured using sucrose the preference test and forced swimming test. The descriptive statistics for sucrose preference rate and immobility time are provided in Table 1, and cumulative distributions of indexes were presented in Fig. 2. Neither sucrose preference rate (Skewness = −0.72, Kurtosis = −0.15) nor immobility time (Skewness = 0.77, Kurtosis = 0.27) were Gaussian distribution. Beta distribution was a better fit for the sucrose preference rate (Shape 1 = 2.53, Shape 2 = 1.08), while Gamma distribution was a better fit for immobility time (Shape = 2.29, Scale = 0.03) tested in treatment native adult rats (Fig. 2).

**Table 1:** Descriptive statistics of readouts of normal rats in behavioral tests

		sucrose preference rate	Immobility time (s)
N		309	309
Mean ± SD		0.70 ± 0.21	86.76 ± 57.28
Skewness		−0.72	0.77
Kurtosis		−0.15	0.27
Percentiles	5	*0.28*	11.45
	10	*0.41*	15.60
	15	0.48	27.55
	25	0.55	46.65
	50	0.73	75.00
	75	0.89	126.50
	85	0.93	*149.40*
	90	0.94	166.60
	95	0.96	*187.95*

### The number of latent classes for behavior of treatment native adult rats

LPA was performed with the indexes of behavioral tests as the indicator variables. Fit indices of the competing latent class models were reported in Table 2. The 4-class and 5-class models were first compared because all the indices suggested that the 4-class model was retained. Although the AIC, BIC, and aBIC were lower in the 5-class models, entropy suggested that the 4-class model was superior to 5-class model. The *P* value of LMR-A for the 5-class model failed to reach statistical significance. Collectively, the 5-class model was not necessarily superior to the 4-class model. Therefore, we rejected the 5-class model and adopted the 4-class model as the one that best fit.

**Table 2:** Fit statistics of competing latent classes

Model	AIC	BIC	aBIC	Entropy	LMR-A (*P*)
1 Class	3306.220	3321.154	3308.467	-	-
2 Classes	3279.370	3305.504	3283.303	0.698	31.045 (0.057)
3 Classes	3258.680	3296.013	3264.297	0.733	25.224 (0.086)
4 Classes	3240.962	3289.495	3248.265	0.754	22.414 (0.019)
5 Classes	3225.607	3285.340	3234.595	0.736	20.182 (0.277)

Table 3 showed the average posterior probability and the number of rats being assigned to a specific latent class. The values on the diagonal ranged from 78.9 to 90.4%, and the values off the diagonal were lower, suggesting a good classification.

**Table 3:** Class assignment probability by class (*N* = 309)

Model	Class-1	Class-2	Class-3	Class-4
	*n* = 44 (14.24%)	*n* = 100 (32.36%)	*n* = 153 (49.51%)	*n* = 12 (14.24%)
Class 1	0.789	0.037	0.175	0.000
Class 2	0.021	0.860	0.098	0.021
Class 3	0.063	0.064	0.873	0.000
Class 4	0.001	0.095	0.000	0.904

### The characteristic of behavior in different classes of adult rats

ANOVA showed that the sucrose preference rates and immobility times were significant differences among the 4 classes. Post-hoc comparisons indicated that rats in class-1 had the lowest sucrose preference rate, followed by class-4, class-2, and class-3. Rats in class-4 had the longest immobility time, followed by class-2, class-1, and class-3 (Table 4). These findings suggested that a latent subgroup of individuals can be identified using the sucrose preference rate and immobility time in untreated adult rats.

**Table 4:** Comparison of behavioral test indexes among four classes

	Class-1	Class-2	Class-3	Class-4	*F*	Post hoc
	*n* = 44	*n* = 100	*n* = 153	*n* = 12		
sucrose preference rate	0.39 ± 0.12	0.74 ± 0.18	0.78 ± 0.14	0.54 ± 0.13	103.05	1 < 4 < 2,3
immobility time	52.18 ± 29.86	136.67 ± 26.61	52.71 ± 23.51	227.77 ± 28.99	355.84	4 > 2 > 1,3

### The operational cutoffs for sucrose preference rate and immobility time

Due to LPA suggesting the existence of latent subgroups with a range of severity of depression in treatment native rats, the percentile method was further used to determine the operational cutoffs based on the skewed distribution of sucrose preference rate and immobility time. The 95-, 90- and 85-percentile (or 5-, 10- and 15-) are used widely for determining reference limits in medicine [12, 14]. In this study, 5- and 10-percentile cutoffs were used to define the anhedonia-like and sub anhedonia-like behavior; 95- and 85-percentile values were used to define the despair-like and sub despair-like behavior. Results showed that the 5-percentile and 10-percentile values of sucrose preference rate were 0.28 and 0.41, respectively (Table 1). This finding means that a sucrose preference rate lower than 0.28 could be defined as anhedonia-like, while a sucrose preference rate between 0.28 and 0.41 could be defined as sub anhedonia-like, and a sucrose preference rate higher than 0.41 could be defined as anhedonia-resilient. Similarly, in the forced swimming test, the 95-percentile of immobility time was 187.95 s, while the 85-percentile was 149.40s (Table 1). This finding suggests that an immobility time longer than 187.95 s could be defined as despair-like, while an immobility time between 149.40s and 187.95 s could be defined as sub despair-like, and an immobility time shorter than 149.40s could be defined as despair-resilient. After escitalopram treatment, rats recovering from depression-like or sub depression-like status (sucrose preference rate > 0.41, immobility time < 149.40s) were defined as recovering, while others were defined as being escitalopram treatment-resilient.

In order to demonstrate that the operational cutoffs in this study are valid, the mean of sucrose preference rate and immobility time in depression-like, sub depression-like, and normal behavior groups were compared. Results showed that there were significant differences in sucrose preference rates among 3 behavioral groups of rats in MD model (F = 109.20, *p* < 0.001), CUS model (F = 172.16, *p* < 0.001), MD + CUS model (F = 291.72, *p* < 0.001), and NOR model (F = 134.59, *p* < 0.001). In addition, there was a significant difference in sucrose preference rates between any two groups of rats in each model (ps < 0.05) (Table 5). Significant difference of immobility times among despair-like rats, sub despair-like rats and despair-resilient rats were also observed in MD model (F = 143.26, *p* < 0.001), CUS model (F = 258.57, *p* < 0.001), MD + CUS model (F = 241.95, *p* < 0.001) and NOR model (F = 196.37, *p* < 0.001). There was a significant difference in immobility time between any two groups of rats in each model (ps < 0.05) (Table 6). These results suggested that the operational cutoffs in this study are sensitive to discriminating anhedonia-like behavior from the sub anhedonia-like, and anhedonia-resilient behaviors induced by stress. Similarly, it can identify despair-like behaviors from sub despair-like and despair-resilient behaviors.

**Table 5:** The sucrose preference rate of stressed rats in sucrose preference test

Models	N	Anhedonia	Sub-anhedonia	Anhedonia-resilience
NOR	120	0.18 ± 0.05^*Δ^	0.35 ± 0.05^*^	0.71 ± 0.16
MD	111	0.20 ± 0.06^*Δ^	0.34 ± 0.06^*^	0.71 ± 0.17
CUS	122	0.21 ± 0.05^*Δ^	0.35 ± 0.06^*^	0.69 ± 0.18
MD + CUS	99	0.19 ± 0.05^*Δ^	0.31 ± 0.06^*^	0.70 ± 0.16

In each model,^*^Compared to Anhedonia-resilience, *p* < 0.05; ^Δ^Compared to sub-anhedonia, *p* < 0.05

**Table 6:** The immobility time of stressed rats in forced swimming test

Models	N	Despair	Sub-despair	Despair-resilience
NOR	120	245.26 ± 30.78^*Δ^	176.68 ± 31.28^*^	87.66 ± 37.94
MD	111	248.91 ± 30.51^*Δ^	172.68 ± 10.07^*^	86.99 ± 39.74
CUS	122	242.30 ± 33.55^*Δ^	173.54 ± 10.41^*^	87.51 ± 39.14
MD + CUS	99	248.82 ± 35.30^*Δ^	176.01 ± 12.58^*^	88.58 ± 36.40

In each models,^*^Compared to despair-resilience, *p* < 0.05; ^Δ^Compared to sub-despair, *p* < 0.05

### The incidence of depression-like and sub depression-like behaviors in CUS-induced rats with and without MD

The sucrose preference test was conducted in MD, CUS, MD + CUS and NOR rats. The mean sucrose preference rates in anhedonia-like, sub anhedonia-like, and anhedonia-resilient rats were not significantly different among MD, CUS, and MD + CUS models. Significant difference in the incidence of anhedonia among 4 models was observed (χ^2^ = 143.24, *p* < 0.001). The incidence of anhedonia-like behaviors in MD + CUS rats (75.76%) was the highest among three models and there was a significant difference between any two models of rats (ps < 0.05). The incidence of sub anhedonia-like behaviors was significantly different among 4 models (χ^2^ = 23.88, *p* < 0.01). The incidence of sub anhedonia-like behaviors in MD + CUS rats (7.07%) was significantly lower than that in MD rats (11.71%), CUS rats (18.85%) (ps < 0.001). However, there was no significant difference in the incidence of sub anhedonia-like behaviors between MD and CUS rats (*p* > 0.05), as well as between MD + CUS and NOR rats (5.00%) (*p* > 0.05). There was a significant difference in incidence of anhedonia-resilient behavior among 4 models (χ^2^ = 142.58, *p* < 0.001). The incidence of anhedonia-resilient behaviors in MD + CUS rats (17.17%) was the lowest and there was a significant difference between any two models of rats (ps < 0.05) (Table 7).

**Table 7:** The incident of anhedonia and sub-anhedonia in stressed rats

	N	Anhedonia n(%)	Sub-anhedonia n(%)	Anhedonia-resilience n(%)
NOR	120	5 (4.17)	6 (5.00)	109 (90.83)
MD	111	19 (17.11)	13 (11.71)	79 (71.17)
CUS	122	50 (40.98)	23 (18.85)	49 (40.16)
MD + CUS	99	75 (75.76)	7 (7.07)	17 (17.17)
χ^2^		143.24	13.88	142.58
p		0.001	0.003	<0.001

The forced swimming test was conducted in MD, CUS, MD + CUS and NOR rats. The mean immobility time in rats with despair-like, sub despair-like, and despair-resilient behaviors were not significantly different among NOR, MD, CUS, and MD + CUS rats. However, the incidence of despair-like behaviors among 4 models was significantly different (χ^2^ = 70.34, *p* < 0.001). The incidence of despair-like behaviors in MD rats (13.51%) was significantly lower than that in CUS rats (39.34%) and MD + CUS rats (43.43%), and higher than that in NOR rats (3.33%) (ps < 0.001). However, there was no significant difference in the incidence of despair-like behaviors between MD + CUS and CUS rats (*p* > 0.05). In contrast, there was significant difference in the incidence of despair-resilient behaviors among the 4 models (χ^2^ = 59.00, *p* < 0.001). The incidence of despair-resilient behaviors in MD rats (73.87%) was significantly higher than that in CUS rats (45.08%) and MD + CUS rats (46.46%), but there was no significant difference in the incidence of sub despair-like behaviors among 4 models (χ^2^ = 1.64, *p* > 0.05) (Table 8). Furthermore, the incidence of stress-resilience was significantly different among the 4 models (χ^2^ = 198.90, *p* < 0.001). The incidence of stress-resilience in MD rats (61.26%) was significantly higher than the incidence of stress-resilience in CUS rats (20.49%) and the incidence of stress-resilience in MD + CUS rats (8.08%) (ps < 0.001). There was also a significantly higher incidence of stress-resilience in CUS rats than the incidence of stress-resilience in MD + CUS rats (*p* < 0.05).

**Table 8:** The incident of despair and sub despair in stressed rats

	N	Despair n(%)	Sub-despair n(%)	Despair-resilience n(%)
NOR	120	4 (3.33)	14 (11.67)	102 (85.00)
MD	111	15 (13.51)	14 (12.61)	82 (73.87)
CUS	122	48 (39.34)	19 (15.57)	55 (45.08)
MD + CUS	99	43 (43.43)	10 (10.10)	46 (46.46)
χ^2^		70.34	1.64	59.00
p		<0.001	0.65	<0.001

### The effect of escitalopram treatment on depression-like and sub depression-like behaviors

Significant difference in recovery rates of anhedonia-like behaviors (the number of animals that no longer exhibited anhedonia behavior) was observed in MD, CUS, MD + CUS and NOR rats after a 4-week escitalopram treatment (χ^2^ = 15.14, *p* < 0.01). Recovery rate of anhedonia-like behaviors in CUS rats (42.00%) was significantly higher than that in MD (21.05%) and MD + CUS rats (12.00%) (ps < 0.01). There was a significantly higher recovery rate of anhedonia-like behaviors in MD rats compared to MD + CUS rats (*p* < 0.01). The recovery rates in rats with sub anhedonia-like behaviors among the 4 models were not significantly different (χ^2^ = 2.35, *p* > 0.05). Furthermore neither the differences of recovery rate in rats with despair-like behaviors (χ^2^ = 0.16, *p* = 0.99) nor in rats with sub despair-like behaviors (χ^2^ = 0.98, *p* = 0.81) were significant different among the 4 models (Table 9).

**Table 9:** The rate of recovery in depressive behaviors after escitalopram treatment

	Anhedonia-like n(%)	Sub anhedonia-like n(%)	Despair-like n(%)	Sub despair-like n(%)
NOR	1 (20.00)	2 (33.33)	3 (75.00)	5 (71.43)
MD	4 (21.05)	4 (30.77)	11 (73.33)	11 (78.57)
CUS	21 (42.00)	12 (52.17)	35 (72.92)	16 (84.21)
MD + CUS	9 (12.00)	2 (28.57)	30 (69.77)	7 (70.00)
χ^2^	15.14	2.35	0.16	0.98
p	0.002	0.50	0.99	0.81

## Discussion

Depression is a highly heterogeneous disease and may be caused by different factors. This requires animal models to validate the casualty between stress and depression that should exclude animals resilient to stresses to mimic the real scenario in human. In addition, the treatment naive animals purchased from commercial resources are generally regarded as behavior normal. However, some animals are much more susceptible to stressors and may have already dropped into an anhedonia and/or despair-like statue during growth and delivery [5], but these animals were not generally excluded from study. It is therefore important to establish a cutoff value to exclude animals resilient to stresses or already having behavioral abnormalities.

In this study, we first performed sucrose preference test and forced swimming test in a large sample of treatment naive adult SD rats. Second, we used the latent profile analysis (LPA) to verify that latent subgroups can be identified in treatment naive adult rats using the sucrose preference rate and immobility time. We found that the naive treatment rats were identified by four classes, which was a low sucrose preference rate and short immobility time in class 1, a high sucrose preference rate and moderate immobility time in class 2, a high sucrose preference rate and short immobility time in class 3, and a moderate sucrose preference rate and long immobility time in class 4, which suggested that the naive adult rats were a heterogeneous group which may mimic the real scenario of heterogeneity in human populations. Third, using the percentile method, we found the existence of a certain natural incidence of depression and resilient to stresses in treatment naive SD rats. As far as we know, this is the first time the percentile method is introduced into an animal model of depression. Due to the stress (Maternal deprivation and/or Chronic unpredictable stress) induced depression-like behaviors were also tested by forced swimming test and/or sucrose preference test in Wistar rats [16, 17] and mice [18, 19]. Thus, the percentile method can also be used to establish cutoff values of depressive behaviors in other animals. Due to different animals, different stresses, and different behavioral test methods can yield different readouts in behavioral tests, a cutoff value depending on the readouts of any behavioral tests lacks a high universality. In contrast, the percentile method has a high universality and can be used in different animals with different behavioral test methods.

Both the clinical and preclinical evidence indicate the existence of subtypes in depression and differences in stresses that may be the main causes leading to the formation of the subtypes [20, 21]. We further tested whether the percentile method has a universality between different stresses (MD vs. CUS), between single and multiple stresses (MD alone, CUS alone, and MD + CUS), and between different subtypes of behaviors (despair-like vs. anhedonia-like). We revealed that 5- and 10-percentile can be used to determine the anhedonia-like and sub anhedonia-like behaviors. While 95- and 85-percentile can be used to determine the cutoffs for despair-like and sub despair-like behaviors. Although significant differences in the mean behavioral readouts between MD, CUS, MD + CUS and NOR rats were observed, the animals’ behaviors underlying three stresses can be grouped as despair-like/anhedonia-like, sub despair-like/sub anhedonia-like, and resilient behaviors using the percentile method. In particular, stress-resilient rats and treatment naive rats responded similar in behavioral tests, which suggests that stress-resilient rats should be subsequently excluded or included according to the purpose of study.

To further elucidate the significance of establishing a cutoff value, we tested the response of rats with different subtypes of behaviors to the current antidepressant escitalopram treatment. The resilient rats were excluded from analysis of escitalopram treatment. Majority of anhedonia-like behaviors were ameliorated by escitalopram treatment. In contrast, both despair-like and sub anhedonic-like behaviors in three models of rats were not reversed by a 4-week escitalopram treatment. Christensen et al. study using Hierarchical clustering analysis revealed a subgroup segregation pattern in the chronic mild stress rat model and antidepressant treatment refractors cluster with anhedonic-like rats. Conversely, stress-resilient rats cluster with rats undergoing anti-depressant-mediated recovery from anhedonia [10]. Thus, establishing a cutoff value to exclude animals resilient to stresses or high sensitive to stresses is also important for preclinical study of new antidepressants.

## Conclusions

In this study, the percentile method is suitable for setting up an operational cutoff for classifying the sensitivity of rats to stresses-induced depression. The significances of the present study included: 1) This study first established a cutoff value in three animal models to study stress-induced depression; 2) Further studies or data analysis with the exclusion of no sensitive animals more accurately reflect the real scenario in humans; 3) Preclinical tests with the exclusion of behavior abnormal animals and resilient animals for therapeutic agents will provide more accurate information, and 4) cutoff value established by the percentile method may have higher universality than if it were established based on the actual readouts of behavioral tests. However, this study only used a single strain of male rats. In the clinic, females are generally more susceptible to depression than males. Moreover, only one model of chronic unpredictable stress and one model of maternal deprivation was used. Currently, there are numerous different experimental models for depression study. Therefore, the generalizability of the results should be considered.
